# Temperature dependent scintillation properties and mechanisms of (PEA)_2_PbBr_4_ single crystals†

**DOI:** 10.1039/d2tc01483a

**Published:** 2022-07-27

**Authors:** Jacob Jasper van Blaaderen, Francesco Maddalena, Cuong Dang, Muhammad Danang Birowosuto, Pieter Dorenbos

**Affiliations:** Delft University of Technology, Faculty of Applied Sciences, Department of Radiation Science and Technology Mekelweg 15 2629 JB Delft The Netherlands J.J.vanBlaaderen@tudelft.nl P.Dorenbos@tudelft.nl; Nanyang Technological University, School of Electrical and Electronic Engineering Nanyang Avenue 50 639798 Singapore; Lukasiewicz Research Network—PORT Polish Center for Technology Development Stablowicka 147 54-066 Wroclaw Poland J.J.vanblaaderen@tudelft.nl

## Abstract

In this work the scintillation properties of PEA_2_PbBr_4_ are studied as function of temperature, accessing the potential use of these materials for low temperature applications. The scintillation properties and mechanism have been studied using a combination of temperature dependent photoluminescence emission and excitation, X-ray excited emission and decay measurements. At room temperature the X-ray excited emission is dominated by the 442 nm emission with a lifetime of 35.2 ns. Under UV-Vis photon excitation an additional emission peak is observed at 412 nm. At 10 K, both X-ray and UV-Vis photon excited emission spectra show a narrow emission peak at 412 nm and a broad emission band centred around 525 nm with a lifetime of 1.53 ns (24%) and 154 ns (76%) respectively. The exact nature of the observed emission peaks is not known. For this reason two potential mechanisms explaining the difference between UV-Vis photon and X-ray excitation and their temperature dependent emissions are explored. The total spectral intensity decreases to 72% of the intensity at room temperature at 10 K. It is suggested that the observed negative thermal quenching behaviour results from a combination of more self absorption and a higher degree of self trapped exciton formation under X-ray excitation. Based on the observed fast decay component at 10 K and light yield of 9400 photons per MeV at room temperature, showing only a 28% decrease at 10 K, could make this material potentially interesting for low temperature and fast timing applications.

## Abstract 2

Low temperature optical and scintillation characterisation of PEA_2_PbBr_4_ single crystals, based on which two potential scintillation mechanisms are proposed.

## Graphical abstract

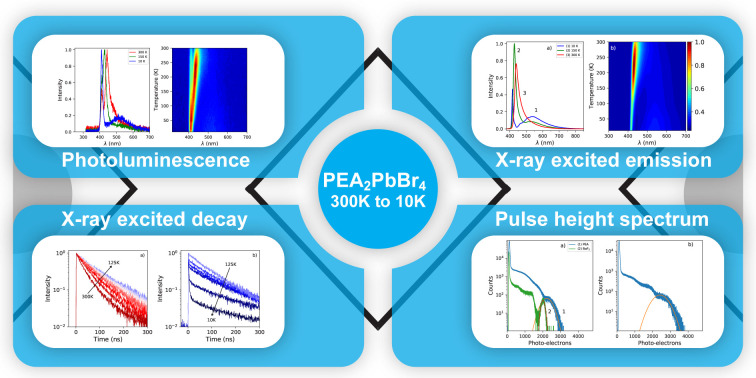

## I. Introduction

In the past two decades, scintillation research has mainly focused on lanthanide activated materials, for example by utilising co-doping strategies.^1–3^ Another less studied class of materials, potentially interesting for scintillation, is wide-band-gap semiconductors. Examples of which are HgI_2_, PbI_2_, and methylammonium lead halides (MAPbX_3_, MA = CH_3_NH_3_, and X = I, Br, or Cl).^4–6^ The latter, belonging to the perovskite family, gained a lot of attention in the early 2010's due to its high efficiency solar cells and optoelectronic applications^7–9^ sparking further interest in the lead-halide perovskite family.

Perovskites can be described by the formula ABX_3_, in which A and B are different size cations, and X an anion.^7^ Crystals can be grown from solution, when A is an organic cation, at relatively low processing temperatures.^10,11^ The presence of heavy Pb atoms in APbX_3_ perovskites makes them very suitable for radiation detection.^12,13^ Typical band-gaps of these materials lay in the range of 1 to 3 eV, significantly increasing their theoretical light yields compared to traditional scintillators.^14^

Experimental evidence of these potential high light yields has been provided by Birowosuto *et al.* and Mykhaylyk *et al.* when studying the cryogenic (7 K) scintillation properties of APbX_3_ perovskites.^15,16^ At room temperature however, a strong decrease in the light yield is observed. This is ascribed to quenching of the exciton luminescence and strong self absorption due to a small Stokes shift.^17,18^

Stabilisation of the exciton luminescence could be achieved by introducing quantum confinement in the system, either by using nano-technology or by changing the 3D perovskite crystals structure. For example, CsPbBr_3_ nano-particles show luminescence at room temperature while bulk crystals do not.^19,20^ An example of such an approach is the implementation of CsPbBr_3_ nano particles in a Cs_4_PbBr_6_ host matrix (CsPbBr_3_ @Cs_4_PbBr_6_).^21^ The potential use of CsPbBr_3_ @Cs_4_PbBr_6_ for scintillation has been discussed elaborately in the recent work of Williams *et al.*,^22^ identifying two main factors prohibiting the use of this material as scintillator: The nano-particle to host energy transfer is not efficient, and the Stokes shift of the exciton luminescence is too small.

There are several ways, as suggested by Wolszczak and Williams *et al.*,^17,22^ in which “The Stokes Shift Problem” could be solved. Both authors identified roughly the same solutions: surrounding the nano particles with wavelength shifting dyes, doping with activator ions, and utilising the formation of self trapped excitons in lower molecular dimension compounds.

One group of lower dimensional materials, from which some show the formation of self trapped excitons,^23–25^ are organic–inorganic lead halide 2D perovskites. They are formed by replacing the A site cation with a large organic molecule, creating a layered structure in which PbX_6_ octahedra are corner connected and separated by a layer of organic molecules. The 2D perovskite phenethylammonium lead bromide (PEA_2_PbBr_4_), is one of the compounds recently gaining interest due to its scintillation properties.^26–28^ It has a density of 2.36 g cm^−3^ and effective atomic number of 32.31.^11^ The light yield was estimated to be approximatly 11.000 photons per MeV (ph per MeV) at room temperature.^29^ Xie *et al.* demonstrated a life time of 11 ns at room temperature, making this material very interesting for fast timing application like in time-of-flight based positron emission tomography (TOF-PET).^30–32^

In this work the scintillation properties of PEA_2_PbBr_4_ are studied as function of temperature. The goal is to asses whether this material is a potentially interesting candidate for low temperature applications, for example TOF-PET. For this PEA_2_PbBr_4_ samples have been studied from 300 K down to 10 K. The samples are studyied using both visible photons and X-rays. The emission spectra recorded using the different means of excitation are compared in order to obtain a better spectroscopic understanding of the processes at hand. A life time study is performed by measuring pulsed X-ray excited decay spectra. The energy resolution and light yield of these samples have been estimated from pulse height spectra, using a ^137^Cs source.

## II. Experimental

PEA_2_PbBr_4_ crystals were synthesised using dimethyl sulfoxide (DMSO, anhydrous), phenylethylammonium bromide ((PEA)Br, 98%), and lead bromide (PbBr_2_, 98%) all were purchased from Sigma-Aldrich and used without further purification. The PEA_2_PbBr_4_ single crystals were synthesised from solution based on the methode described and used by Maddalena *et al.*^29^ A 3 M precursor solution of (PEA)Br and PbBr_2_, in stoichiometric amounts, was dissolved in DMSO and mixed at 100 °C for 2 h under a N_2_-atmosphere. Afterwards, the solution was left to evaporate at room temperature in open air for at least 1 week up to one month until large crystals formed in the liquid. The crystal precipitate was filtered from the solution, washed with hexane and dried under vacuum for future characterisation. This resulted in flat transparent crystals of approximately the following dimensions: 8 mm × 5 mm × 1 mm. Unless mentioned otherwise a crystal of this size was used.

All X-ray diffraction spectra are recorded on a Bruker D8 Discover (Cu Kα (*λ* = 1.54 Å)). A step size and integration time of 0.02° and 1 second respectively. The XRD spectrum is shown in Fig. S1 (ESI†).

All photoluminescence emission and excitation spectra are recorded using light from a 450 W Xenon lamp passing through a Horiba Gemini 180 monochromator. The emission light passes through a long pass filter and Princeton Instruments SpectraPro-SP2358 monochromator, and is detected by a Hamamatsu R7600U-20 PMT. All recorded spectra are corrected for the lamp intensity. A single crystal was used for all photoluminescence measurements. The crystals were mounted on the cold finger of a closed cycle helium cryostat operating below 10^−4^ mbar. Unless mentioned otherwise all photoluminescence emission spectra are recorded in reflectivity mode.

All absorption spectra are recorded using a PerkinElmer Lambda 1050. The precursor salt was dissolved in ethanol; the measurement was performed using a quartz cuvet.

X-Ray excited luminescence spectra were recorded by exciting the samples with X-rays from an tungsten anode X-ray tube operating at 79 kV resulting in an average X-ray energy of 40 keV. A filter was used to remove the lower energy side of the produced X-ray spectrum in order to prevent radiation damage of the sample. A single crystal was mounted on the cold finger of a closed cycle helium cryostat operating below 10^−4^ mbar. The emission light was collected by a parabolic mirror and coupled into an optical fibre. The spectrum was recorded by an Ocean Insights QE Pro Spectrometer. The parabolic mirror is positioned under a 90 degree angle compared to the X-ray tube.

X-Ray excited decay spectra were recorded using the time-correlated single photon counting method. A PicoQuant LDH-P-C-440M pulsed diode laser is used to excite a Hamamatsu N5084 light excited X-ray tube producing X-ray with an average energy of 18.5 keV. The laser head is triggered by a Picoquant laser driver. The reference output of the driver is used as start input and is connected to an Ortec 567 time-to-amplitude converter (TAC). The bin width of the latter was calibrated using an Ortec 462 time calibrator. The emitted photons are recorded using an ID Quantique id100-50 single-photon counter who's output signal was used as the stop input of the TAC. The signal first goes through a LeCroy 623B Octal Discriminator and analogue delay. The start and stop time differences are collected and digitised using an Ortec AD114 amplitude-to-digital converter. All temperature dependent measurements were performed using a closed cycle helium cryostat operating below 10^−4^ mbar.

Pulse height spectra were recorded by mounting the samples on a Hamamatsu R1791 PMT covered with PTFE tape operating at −700 V. The signal passes through an integrated pre-amplifier and Ortec 672 spectroscopic amplifier before it is processed by an Ortec AD114 26 K ADC. All measurements were performed without optical coupling, the samples were directly mounted on the entrance window.

## III. Results

### A. Spectroscopy

**Fig. 1:**
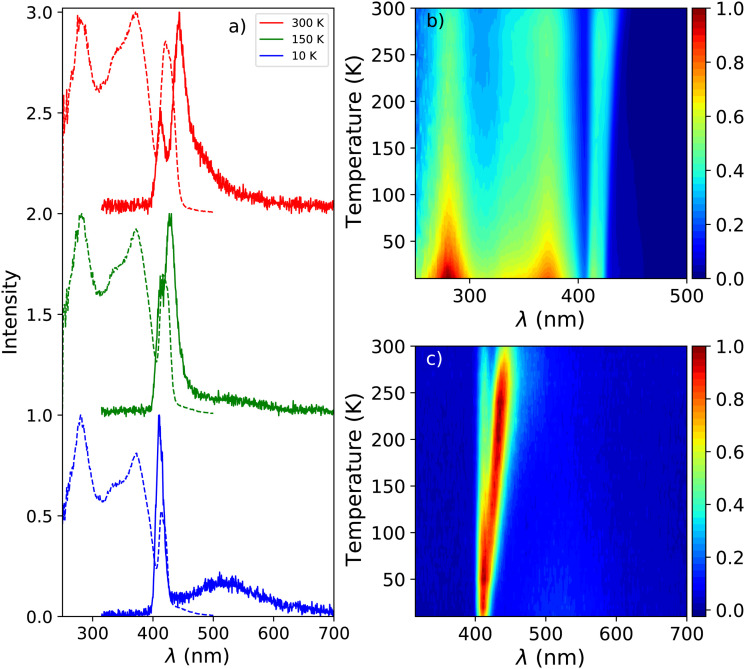
(a) Temperature dependent photoluminescence emission (*λ*_ex_ = 260 nm) and excitation (*λ*_em_ = 530 nm) spectra of PEA_2_PbBr_4_, from top to bottom: 300 K, 150 K, and 10 K. (b) Temperature dependent photoluminescence excitation spectra (*λ*_em_ = 530 nm), from 300 K down to 10 K. (c) Temperature dependent photoluminescence emission spectra (*λ*_ex_ = 260 nm), from 300 K down to 10 K.

Fig. 1a shows the photoluminescence emission (PL) and excitation (PLE) spectra of PEA_2_PbBr_4_ at 300 K, 150 K, and 10 K. An overview of the spectral change upon cooling from 300 K down to 10 K is shown in Fig. 1b and c. The emission spectrum recorded at 300 K shows two peaks, centred around 412 nm and 442 nm. On the longer wavelength side of the latter, a long tail is observed. Upon cooling a blue shift is observed for the 442 nm emission, eventually merging with the 412 nm emission. This shift is clearly visible in the PL spectrum recorded at 150 K, shown in Fig. 1a. Additionally, a broad emission band centred around 525 nm appears at low temperature. The 412 nm emission peak is observed both by Kawano *et al.* in PEA_2_PbBr_4_ single crystals and Guo *et al.* in PEA_2_PbBr_4_ nano platelets.^26,33^ Both ascribe it to free exciton emission from the inorganic Pb–Br layer. Kawano *et al.* ascribe the 442 nm emission peak to lattice defects creating donor and acceptor states close to the band edges.^26^ The broad 525 nm emission band, observed at low temperatures, is ascribed to the formation of self trapped excitons.^34^ The same assignments will be used in this work.

**Fig. 2:**
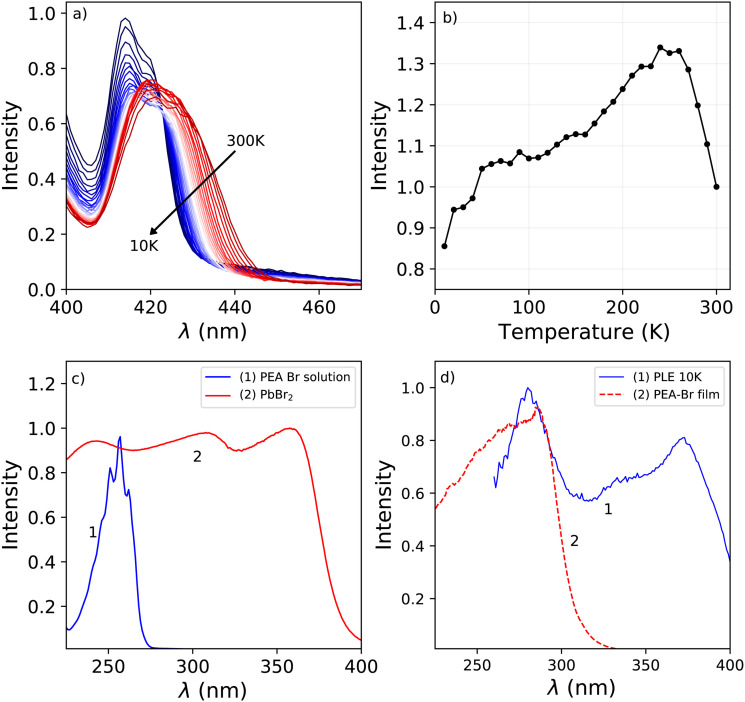
(a) Temperature dependent photoluminescence excitation spectra, showing only the 423 nm excitation band. Temperature decreases in the direction of the arrow. (b) Change of the total spectral photoluminescence emission intensity as function of temperature. (c) Comparison between (1) the absorbance spectrum of PEA-Br salt dissolved in ethanol, and (2) the absorbance spectrum of PbBr_2_ powder. (d) Comparison between (1) the 280 nm band from the 10 K PLE spectrum, and (2) the absorbance spectrom of a film of PEA-Br.

The excitation spectrum recorded at 300 K, shown in Fig. 1b, contains three distinct bands centred around 423 nm, 370 nm with a shoulder on the shorter wavelength side, and 280 nm. The spectra are recorded in the shoulder of the 442 nm emission to reveal the spectral overlap between the PL and PLE spectra. No significant changes where observed when recording the PLE spectra at the emission maxima. The first two bands, 423 nm and 370 nm, are ascribed to the formation of excitons and across band gap transitions respectively.^35,36^ Additionally, as can be seen in Fig. 2a, a second peak is observed in the 423 nm band, suggesting that the observed band could be a combination of the often observed exciton peak and absorption of lattice defects. Upon cooling, the latter shows a blue shift to 413 nm at 10 K, the other bands do not show a wavelength shift, as can be observed in Fig. 1b. The blue shift of the 423 nm excitation band can also be observed in Fig. 1a. As a result is shows more overlap with the 410 nm emission at 10 K compared to 300 K.

The absorbance spectra of the PEA-Br precursor, dissolved in ethanol, and PbBr_2_ precursor in powder form are shown in Fig. 2c. The peak observed in the absorbance spectrum of the dissolved PEA-Br precursor salt shows a clear fine structure at the same positions as the different transitions observed for liquid benzene.^37^ This same structure is observed, although in a lesser degree, in the band centred around 280 nm in the 10 K excitation spectrum shown in Fig. 2d. The latter shows the absorbance spectrum of a film of PEA-Br precursors and the 280 nm band from the 10 K PLE spectrum. Both show significant overlap, suggesting that the absorption wavelength of the PEA-precursor shifts by changing its local surroundings. Based on these spectra, Fig. 2c and d, the 280 nm band is ascribed to absorption from the organic layer.

The total integrated spectral photoluminescence emission intensity is shown in Fig. 2b. Upon cooling the total intensity starts to increase, reaching its maximum around 250 K. Below this temperature the intensity decreases, reaching 82% of the intensity at 300 K. Based on the temperature dependent PL spectra, shown in Fig. 1c, it can be concluded that the initial intensity increase originates from the 442 nm emission. The decrease of the intensity below 250 K looks similar to the behaviour observed by Xie *et al.* and Maddalena *et al.* upon integration of their X-ray excited emission spectra of PEA_2_PbBr_4_.^29,32^ Such behaviour has also been observed for n-type GaAs and n-type ZnS and is referred to as negative thermal quenching.^38^ However, under X-ray excitation Xie *et al.* observed a minimum in the integrated intensity at 190 K, after which the intensity increases again, reaching a plateau value of approximately 25% of the intensity measured at 350 K. This behaviour is not observed under photon excitation, for which only a decrease with intensity is observed, see Fig. 2b.

### B. X-Ray excited luminescence

**Fig. 3:**
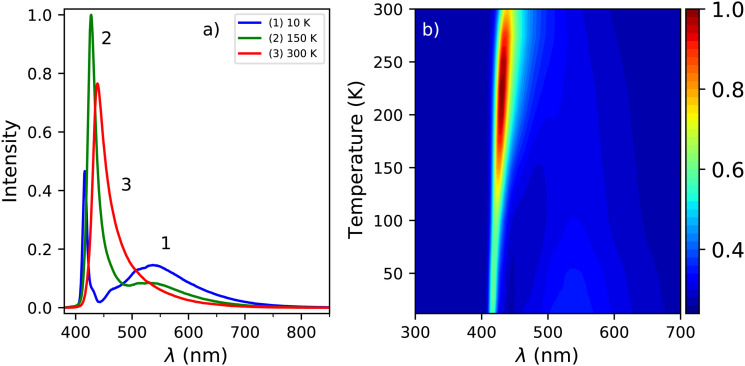
(a) X-ray excited emission spectra at 300 K, 150 K, and 10 K. (b) Temperature dependent X-ray excited emission spectra from 300 K down to 10 K.

The X-ray excited emission spectra of PEA_2_PbBr_4_ at 300 K, 150 K, and 10 K are shown in Fig. 3a. Only one emission peak, centred around 440 nm, is observed at 300 K. Similar results were obtained by van Eijk *et al.* and Maddalena *et al.*^27,29^ Based on the PL spectrum recorded at 300 K, shown in Fig. 1a, this peak is ascribed to lattice defects creating donor and acceptor states close to the band edges. At 10 K a narrow peak centred around 414 nm and a broad emission band centred around 550 nm are observed. Similar behaviour was observed by Xie *et al.* and Maddalena *et al.*^29,32^ The 414 nm and 550 nm peaks are ascribed to free exciton and self trapped exciton emission. This is based on the 10 K PL spectrum shown in Fig. 1a. An overview of the spectral change upon cooling from 300 K to 10 K is shown in Fig. 3b, clearly demonstrating the blue shift of the 440 nm peak and development of the broad 550 nm emission band at low temperature which can also be observed in Fig. 3a.

**Fig. 4:**
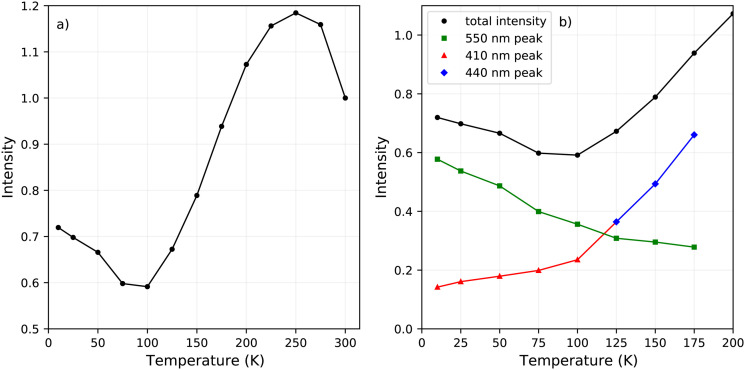
(a) Change of the total spectral intensity as function of temperature upon X-ray excitation. (b) Change of the total spectral intensity and contributions from the 410 nm, 440 nm, and 550 nm peaks between 200 and 10 K.

The change in the total spectral intensity of the X-ray excited emission spectra as function of temperature is shown in Fig. 4a. At 10 K the intensity has decreased to 72% of the total integrated intensity at 300 K. Similarly to the total spectral photoluminescence intensity, shown in Fig. 2a, an initial increase of the intensity is observed upon cooling which was ascribed to the temperature behaviour of the 442 nm band. The decrease after 250 K, with a minimum at 100 K, looks similar to the total integrated spectral intensity already observed for X-ray excited emission spectra of PEA_2_PbBr_4_.^29,32^

The contribution of the different emission bands to the total spectral intensity under X-ray excitation is shown in Fig. 4b, focusing on the range between 10 and 200 K. Below 125 K, the blue shift of the 442 nm emission stops to become completely merged with the 412 nm emission. Both show a decrease in intensity upon cooling. The broad 550 nm emission, ascribed to self trapped exciton formation, shows an increase in its spectral intensity upon cooling resulting in the typical negative thermal quenching behaviour of PEA_2_PbBr_4_.

### C. X-Ray excited decay

**Fig. 5:**
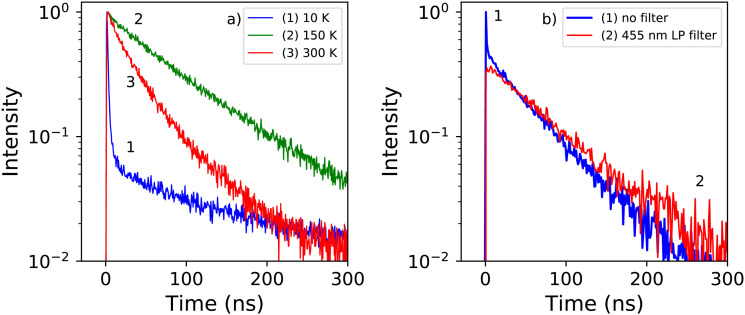
(a) Temperature dependent X-ray excited decay spectra of PEA_2_PbBr_4_ recorded at 10 K, 75 K, and 300 K. (b) X-ray excited decay spectra of PEA_2_PbBr_4_ recorded at 10 K with and without 455 nm long pas filter.

Fig. 5a shows the X-ray excited decay spectra of PEA_2_PbBr_4_ at 10 K, 150 K, and 300 K. The fits to these spectra are shown in Fig. S2 (ESI†). At 300 K only one decay component is observed with a lifetime of 35.2 ns. This is significantly slower compared to the 11 ns decay time measured under 662 keV gamma excitation as reported by Xie *et al.*^32^ At 150 K the lifetime has increased to 90 ns. Based on the X-ray excited emission spectra shown in Fig. 3a it is suggested that the decay between 300 K and 125 K originates from the emission band centred around 440 nm. At 10 K the decay spectrum changes significantly, showing two decay components. A fast component with a life time of 1.53 ns, and a slow component with a life time of 154 ns. Similar behaviour was observed by van Eijk *et al.* at 100 K.^27^ The fitted curves and obtained fitting parameters are shown in Fig. S2 and Table S1 (ESI†) respectively. From the latter it is estimated that the intensity of the 1.54 ns component is 6 times smaller compared to the 154 ns component.

In order to distinguish the origin of the two components, the decay spectrum are recorded using a 455 nm long pass filter, separating the narrow and broad emission bands observed in the 10 K X-ray excited emission spectrum, see Fig. 3a. The resulting decay spectra are shown in Fig. 5b. At 10 K the fast component is no longer present when the long pass filter is placed in front of the detector. This suggests that the fast decay component at 10 K originates from the 414 nm emission, and the slow decay component from the 550 nm emission.

**Fig. 6:**
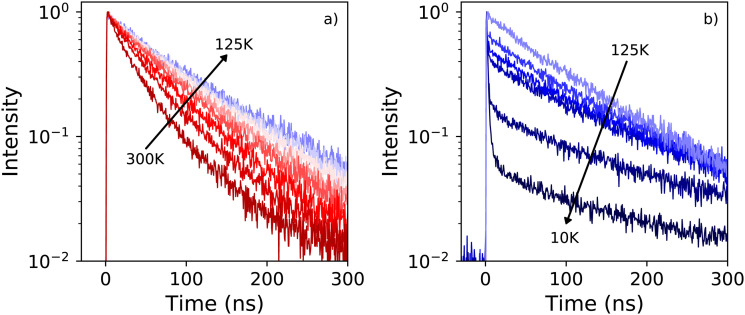
(a) Temperature dependent X-ray excited decay spectra of PEA_2_PbBr_4_ recorded from 125 K to 300 K, with a 25 K interval. The temperature decreases in the direction of the arrow. (b) Temperature dependent X-ray excited decay spectra of PEA_2_PbBr_4_ recorded from 125 K to 25 K, with a 25 K interval, and at 10 K. The temperature decreases in the direction of the arrow.

**Fig. 7:**
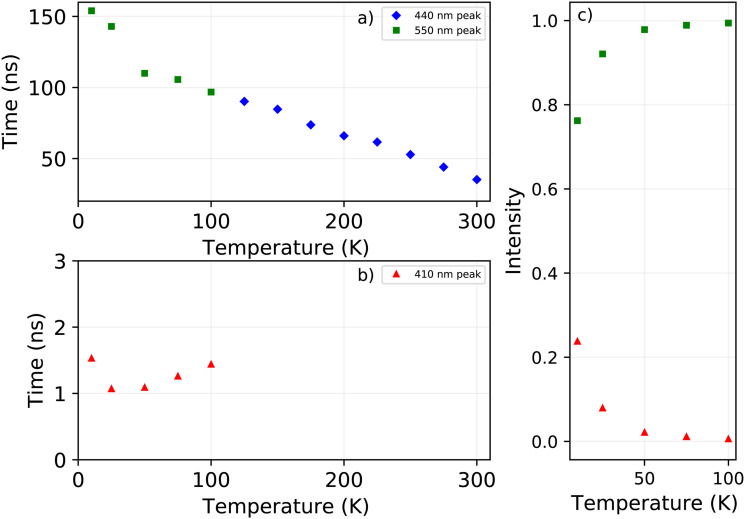
Temperature dependent decay times, obtained from X-ray excited decay spectra (a) for the 440 nm and 550 decay components (b) for the 410 nm decay component. (c) Intensity of the 410 nm and 550 nm decay components between 10 K and 100 K.

The X-ray excited decay spectra measured as function of temperature are shown in Fig. 6a and b. Upon cooling two different regimes can be identified. An increase of the life time is observed when cooling from 300 K to 125 K, as can be observed in Fig. 7. The direct comparison of the latter in Fig. 6a clearly demonstrates the non-exponential form of the decay spectrum at 300 K. At lower temperatures the decay spectrum looks more like a single exponential. The temperature range from 125 K down to 10 K is shown in Fig. 6b, revealing the development of the fast decay component below 125 K. This temperature corresponds to the moment the blue shift of the 442 nm emission band stops, as can clearly be seen in Fig. 1c, leaving only the 412 nm and 550 nm emissions. The life time of the 550 nm emission follows a trend similar to the life time of the 440 nm emission, see Fig. 7. The lifetime of the 412 nm emission becomes faster upon cooling, interestingly showing an increase at 10 K, see Fig. 7.

### D. Pulse height spectroscopy

**Fig. 8:**
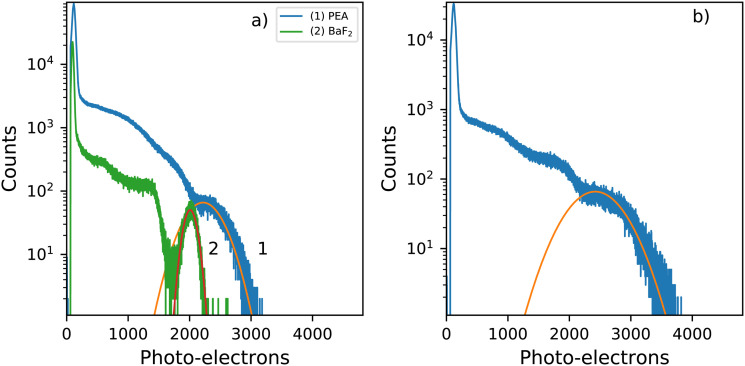
Pulse height spectra recorded using a ^137^Cs γ-source and a PMT as detector (a) Small, similar sized, PEA_2_PbBr_4_ and BaF_2_. (2 mm × 1 mm × 0.3 mm) (b) Large PEA_2_PbBr_4_ crystal (8 mm × 5 mm × 1 mm).

Fig. 8a shows the ^137^Cs pulse height spectra of a small PEA_2_PbBr_4_ crystal (2 mm × 1 mm × 0.3 mm). The spectrum is recorded using a PMT and shaping time of 0.5 μs. From this pulse height spectrum, a light yield of 8600 ph per MeV and energy resolution of 29% are estimated. Similar values were reported by van Eijk *et al.*^27^ The light yields were estimated based on the method described by de Haas and Dorenbos.^39^ The observed photo peak is significantly broadened due to escape of the 75 keV characteristic K X-rays of lead. The pulse height spectrum of BaF_2_ is added as a reference crystal, of similar size, for which a light yield of 7800 ph per MeV and energy resolution of 11% are estimated. The characteristic X-rays of barium are of significantly lower energy 32.2 keV K X-rays, compared to those of lead decreasing the broadening effect, hence inproving the energy resolution.

Fig. 8b shows the pulse height spectrum of the large crystal (8 mm × 5 mm × 1 mm), the same sample is used in the decay measurements and X-ray excited emission measurements. The spectrum is recorded using a PMT and shaping time of 0.5 μs. For this sample a light yield of 9400 ph per MeV and energy resolution of 39% are estimated based on the pulse height spectrum. The total spectral intensity of this sample decreases to 72% of the room temperature intensity uppon cooling down to 10 K, as can be observed in Fig. 4a.

## IV. Discussion

### A. Wavelength shifts, emission, and excitation spectra

Fig. 1b and c show the photoluminescence emission and excitation spectra as function of temperature. The 442 nm emission peak and 423 nm excitation band both show a blue shift upon cooling. The 410 nm emission peak and the excitation bands centred around 370 nm and 280 nm, however, do not. For this reason it is suggested that the observed blue shift of the aforementioned peaks does not result from changes in the band gap as function of temperature. If this would be the case one would expect all emissions to show a wavelength shift. The assignment of the 442 nm emission to bound exciton emission is based on the work of Kawano *et al.*^26^ However, based on the observed behaviour of the 442 nm emission as function of temperature this might not be correct. Based on the spectra shown in Fig. 1a is could also suggest that the peak observed at 412 nm is artificial, resulting from self absorption. A better understanding of the nature of the observed emissions is needed in order to understand the observed temperature dependent behaviour.

### B. X-Ray *versus* UV-Vis photon excited emission

Fig. 1a and 3a show the photoluminescence emission spectrum and X-ray excited emission spectrum of PEA_2_PbBr_4_ recorded at 300 K. The main difference is the absence of the 412 nm emission peak, ascribed to free exciton emission, in the X-ray excited spectrum. The X-ray attenuation length is estimated to be 560 μm, based on the linear attenuation coefficient shown in Fig. S3 (ESI†) and the average X-ray energy of 40 keV. The samples, as mentioned in the experimental section, have a thickness of 1 mm. Most of the X-ray energy will thus be deposited in the bulk of the crystal, the rest will escape at the back.

From Fig. 1a it can be observed that the 412 nm emission peak completely overlaps with the 423 nm excitation peak at 300 K. The overlap does not change upon cooling. This suggests that the 412 nm emission could potentially not be visible under X-ray excitation due to self absorption, but would still be present under UV-Vis excitation due to the difference in penetration depth. This would allow the 412 nm emission to escape at the surface of the crystal before being reabsorbed. The influence of self absorption can be studied by measuring the room temperature PL spectrum in reflectivity and transmission mode. When measuring in reflectivity mode the excitation source and detector are placed at a 90 degree angle. When measuring in transmission mode the excitation source and detector are facing each other, forcing the emission light to pass through the sample before it is detected. The resulting spectra are shown in Fig. S4 (ESI†). The 412 nm emission is no longer present when measuring in transmission mode, confirming the presence of self absorption.

Alternatively, one might suggest that the difference is caused from the formation of spatially separated charge carriers created upon X-ray excitation. Before emission, the spatially separated electrons and holes need to approach each other first, before forming excitions. This could allow them to relax to ether lattice defects forming the 442 nm emission in stead of free excition emission or for one of the spatially separated charge carriers to self trap contributing to the higher self trapped exciton emission under X-ray excitation. The data presented in this paper does not provide a conclusive argument to distinguish between the mechanisms discussed above.

### C. Negative thermal quenching

Fig. 2a and 4a show the total integrated intensity upon photon excitation and X-ray excitation respectively. Both show an initial intensity increase upon cooling, ascribed to the 442 emission peak, which is rather similar to normal thermal quenching behaviour reaching its maximum at 250 K. Below this temperature both show an intensity decrease, referred to as negative thermal quenching. Under X-ray excitation an intensity minimum is reached at 100 K, after which the intensity increases again reaching a plateau value of 72% compared to the intensity at 300 K. This behaviour is not observed under photon excitation.

Fig. 4b shows the contribution of the different emissions to the total spectral intensity upon X-ray excitation. This Figure nicely demonstrates that the negative thermal quenching behaviour is the result of the self trapped exciton emission increasing in intensity upon cooling, while the other emissions decrease in intensity. Upon comparing the PL spectra at 10 K and X-ray exited emission spectrum at 10 K, a direct comparison is shown in Fig. S5, (ESI†) it can be observed that the self trapped exciton emission increases under X-ray excitation. The total spectral intensity under UV-Vis excitation, shown in 2a, does not show an increase below 100 K. The observed difference in the negative thermal quenching behaviour under X-ray and UV-Vis photon excitation is ascribed to the different intensities of the self trapped exciton emission.

## V. Conclusion

The potential of PEA_2_PbBr_4_ for low temperature scintillation applications has been accessed. The room temperature emission, under photo excitation, consists of two emissions ascribed to free excitons and excitons bound to lattice defects. Only the latter is observed under X-ray excitation. The precise nature of the emissions, however, is not known thus making it difficult properly explain the temperature dependent photoluminescence behaviour. At 10 K only free exciton emission and self trapped exciton emission are observed, for both photo and X-ray excitation. The intensity of the self trapped exciton emission is larger under X-ray excitation. The observed negative thermal quenching behaviour observed for PEA_2_PbBr_4_, is ascribed to a combination of self absorption and self trapped exciton emission competing with the other emissions.

X-Ray excited decay spectra have demonstrated that, below 125 K, the decay consists of two components. A fast component ascribed to the free exciton emission and a slow component ascribed to self trapped exciton emission, having a life time of 1.53 ns (24%) and 154 ns (76%) at 10 K. The total spectral intensity, under X-ray excitation, only decreases to 72% at 10 K. The combination of the fast decay component and decent light yield make this material interesting for low temperature and fast timing applications.

## Author contributions

J. J. B. was responsible for performing all experiment and writing part of the manuscript; F. M. and C. D. were responsible for the synthesised perovskite crystals, reviewing and editing the manuscript; M. D. B. was responsible for the supervision of the project, writing part of the manuscript, and reviewing and editing the manuscript; P. D. was responsible for the supervision of the project, and reviewing and editing the manuscript. All authors have given approval to the final version of the manuscript.

## Conflicts of interest

There are no conflicts to declare.

## Supplementary Material

TC-010-D2TC01483A-s001
