# Supplementary material for: Temperature dependent scintillation properties and mechanisms of (PEA)2PbBr4 single crystals
Source: J Mater Chem C Mater. 2022 Jul 27;10(32):11598–606. doi: 10.1039/d2tc01483a (PMC9386685; doi:10.1039/d2tc01483a)
Supplement: TC-010-D2TC01483A-s001 [file TC-010-D2TC01483A-s001.pdf]

Electronic Supplementary Material (ESI) for Journal of Materials Chemistry C.
This journal is © The Royal Society of Chemistry 2022

Temperature Dependent Scintillation Properties and Mechanisms of $(PEA)_2PbBr_4$ Single Crystals

Supplementary Information

Jacob Jasper van Blaaderen[1], Francesco Maddalena[2], Cuong Dang[2], Muhammad Danang Birowosuto[3], Pieter Dorenbos[1]

1: Delft University of Technology,
Faculty of Applied Sciences,
Department of Radiation Science and Technology,
Mekelweg 15, 2629 JB Delft, Netherlands

2: Nanyang Technological University,
School of Electrical and Electronic Engineering,
Nanyang Avenue 50,
639798, Singapore, Singapore

3: Lukasiewicz Research Network—PORT Polish Center for Technology Development,
Stablowicka 147, 54-066 Wroclaw, Poland
email: J.J.vanblaaderen@tudelft.nl

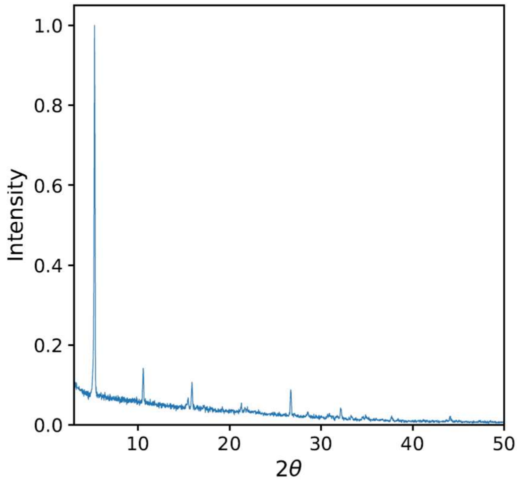

Figure S1: Powder XRD spectrum $(PEA)_2PbBr_4$.

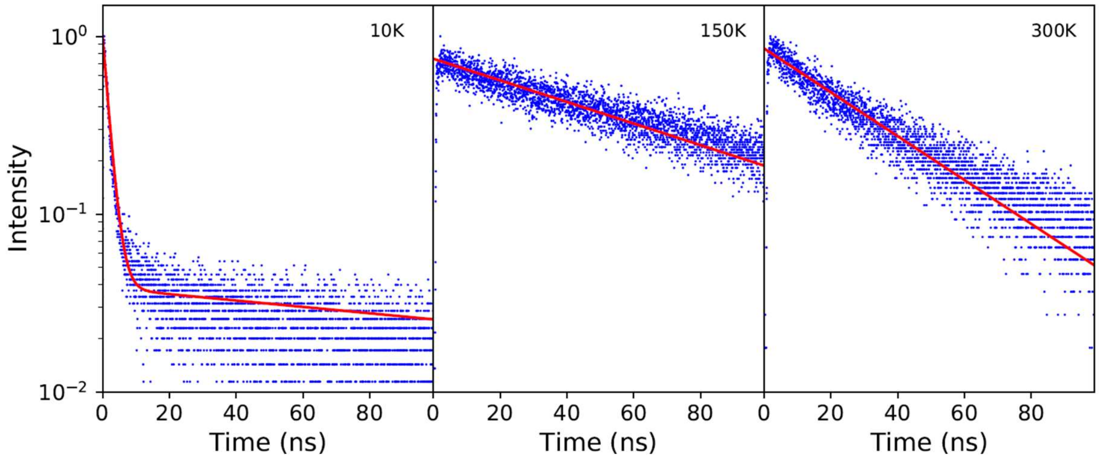

Figure S2: Fitted decay spectra, from left to right, at 10K, 150K, and 300K.

Table S1: Parameters obtained from fitting the decay spectra shown in Figure S3

	$\tau_{440nm}(ns)$	I_{440nm}	$\tau_{550nm}(ns)$	I_{550nm}	$\tau_{410nm}(ns)$	I_{410nm}
300K	35.2	0.855				
150K	90.2	0.748				
10K			154	0.0385	1.53	1.21

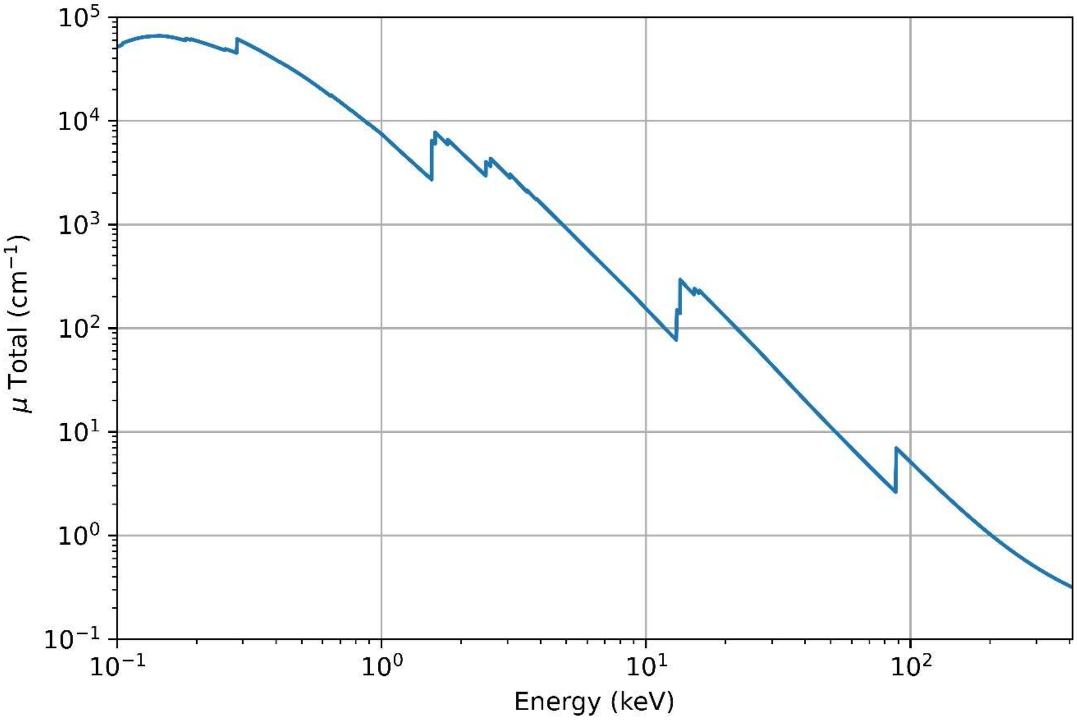

Figure S3: Linear attenuation coefficient PEA_2PbBr_4.

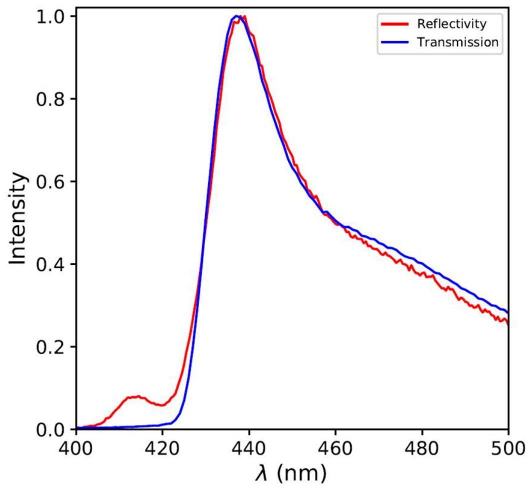

Figure S4: Comparison of the photoluminescence spectra measured in reflectivity mode and transmission mode.

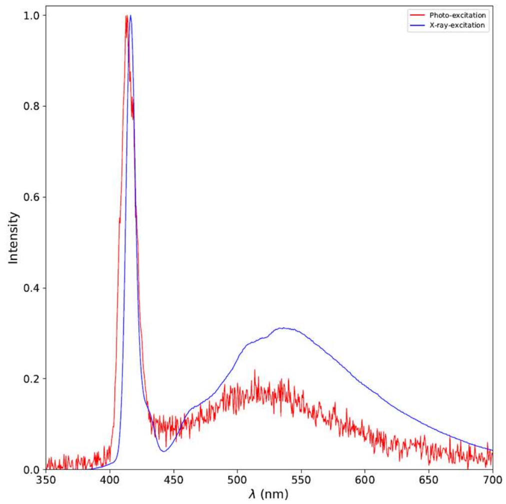

Figure S5: Comparison of the photoluminescence emission and x-ray excited emission spectra recorded at 10K.
